# Gastrointestinal function development and microbiota

**DOI:** 10.1186/1824-7288-39-15

**Published:** 2013-02-24

**Authors:** Antonio Di Mauro, Josef Neu, Giuseppe Riezzo, Francesco Raimondi, Domenico Martinelli, Ruggiero Francavilla, Flavia Indrio

**Affiliations:** 1 Department of Pediatrics, University of Bari, Policlinico, Piazza G Cesare, Bari, Italy; 2 University of Florida, Gainesville, FL, USA; 3 Lab of Experimental Pathophysiology, IRCCS Castellana Grotte, Castellana Grotte, Italy; 4 University Federico II, Naples, Italy

**Keywords:** Intestinal microbiota, Feeding intolerance, Microbial effect on gastrointestinal function development

## Abstract

The intestinal microbiota plays an important role in the development of post-natal gastrointestinal functions of the host. Recent advances in our capability to identify microbes and their function offer exciting opportunities to evaluate the complex cross talk between microbiota, intestinal barrier, immune system and the gut-brain axis. This review summarizes these interactions in the early colonization of gastrointestinal tract with a major focus on the role of intestinal microbiota in the pathogenesis of feeding intolerance in preterm newborn. The potential benefit of early probiotic supplementation opens new perspectives in case of altered intestinal colonization at birth as preventive and therapeutic agents.

## Introduction

In the human gut resides the *microbiota*, a large and diverse community of microorganism, dominated by bacteria, known to have a critical role in the evolution of the intestinal functions and in overall health of the host [1].

The bacterial cells far outnumber the human cells of the host that harbors them and the total amount of genes in the various species represented in our indigenous microbial communities is estimated about 2–4 million, exceeding the number of our human genes >100-fold [2]. Through expression of this exceptional quantity of genes, whose totality is termed the “*microbiome*”, intestinal bacteria can execute numerous enzymatic reactions that the mammalian host is not able to catalyze. This is the reason why the microbiota is now considered as an “organ within an organ”, with its own functions: it modulates expression of genes involved in mucosal barrier fortification, angiogenesis and postnatal intestinal maturation. It also has a critical role in supporting normal digestion and affects energy harvest from the diet and energy storage in the host, fermenting unused energy substrates to short chain fatty acids (SCFAs) [3].

Microbial functions are intimately strain-related and even different strains of a single species may differ in the effects they produce.

Primary colonization of the gut can be regarded as an important stage of development of intestinal functions and the transference of the microbiota at birth from maternal vaginal and intestinal flora comprises a hereditary succession of a parallel genome [4].

A large interface between the external environment and the mammalian host is represented by the intestinal epithelium. The complex cross-talk between the gut and its microbial content is a normal part of development and plays a determinant role in the capacity to distinguish potentially dangerous from harmless bacterial and food antigens. This function requires sophisticated sensor systems to be responsive to a wide variety of microbial and food antigens that transits or populates the GI tract [5].

The intestinal microbiota possesses an immunomodulatory capacity, affecting a variety of signaling pathways with modulation of proper immune, inflammatory and allergic responses. An imbalance of normal intestinal microbiota, or the host response to such an imbalance are considered to be involved in the pathogenesis of a variety of intestinal disorders [6].

### Intestinal colonization

Newborn infants exit the uterus and enter an extrauterine environment filled with microbes. The gastrointestinal tract of a normal fetus is generally thought to be sterile. However, recent studies using molecular techniques are suggesting that the fetal intestine may be exposed to microbes via swallowing of colonized amniotic fluid [7,8]. Occult microbes in amniotic fluid may be associated with preterm labor and premature rupture of membranes.

Despite ongoing studies to determine the qualitative and the quantitative state of microorganisms in amniotic fluid, this new aspect of fetal gut colonization remains a largely unexplored area. The finding of microbial DNA in meconium of preterm infants offers the opportunity to further explore the intraamniotic microbial milieu of newly born infants [9].

During delivery, bacteria from maternal vaginal and intestinal microbiota in vaginal birth or from maternal skin surface and the surrounding environment in cesarean section, colonize the gut of the newborn with different microbial strains [10]. A great number of bacteria, both beneficial and harmful, can colonize the gastrointestinal tract. Some bacteria family are common pathogens, such as *Clostridiacea, Pseudomonadaceae* and *Staphylococcaceae*.Others can be either pathogenic or beneficial such as *Bacterioidaceae* and *Enterobacteriaceae*. Still others are thought to be primarily beneficial, most commonly *Lactobacillaceae* and *Bifidobacteriaceae*.

Although vary studies have been performed to sample the general composition of the infant gut, the real microbial biodiversity still remain controversial and unknown; caution must be applied in the interpretation of the different results obtained by both culture-dependent and metagenomic techniques, due to technical biases [11].

According to delivery mode, caesarean section delivered newborns, that are deprived of contact with maternal vaginal microbiota, have a deficiency of strict anaerobes with lower numbers of *E. coli*, *Bacteroides* and Bifidobacteria and an higher presence of facultative anaerobes such as *Clostridium* species, compared with vaginally born infants [12].

Also infant’s gestational age at birth seem to have significant effects on the intestinal microbiota [13]. It’s now clear that the pattern of bacterial colonization in the preterm neonatal gut differs from that in the healthy, full-term neonatal gut. This aberrant colonization, mostly due to the routine use of sterile formula and antibiotics in neonatal intensive care unit (NICU), could have a central role in feeding intolerance and in development of necrotizing enterocolitis (NEC), a devasting disease affecting primarily premature infants [14].

Afterwards, in the early stage of life, the composition of the intestinal microbiota undergoes major modifications, mostly influenced by the feeding pattern [10]. Gastrointestinal flora composition differs substantially in breast-fed infants and formula-fed infants because of the differences in composition between human milk and standard infant formula. For example, breast milk-fed infant microbiota is composed by an increased number of bifidobacteria and *lactobacilli*, whereas formula-fed infant microbiota has more *enterococci* and *enterobacteria*. This difference is thought to be due to the breast milk composition of molecules with antimicrobial activity and prebiotic oligosaccharides, thought to have a beneficial role for the infant [15,16]. Furthermore, there is accumulating evidence that human milk is not sterile but contains maternal derived bacterial molecular motifs that are thought to influence the newborn’s immune system development. This procedure is called “bacterial imprinting”, and its overall biological effect requires further study [17,18].

The initial bacterial colonization after birth, and its change according to environment, nursing, weaning and drugs, plays a crucial function in the final development of the gut with large shifts in the relative abundances of taxonomic groups. The composition of microbiota undergoes significant changes in infancy. Some authors observed a gradual increase in diversity over time, with a discrete steps of bacterial succession according to similar life events [19].

**Figure 1:**
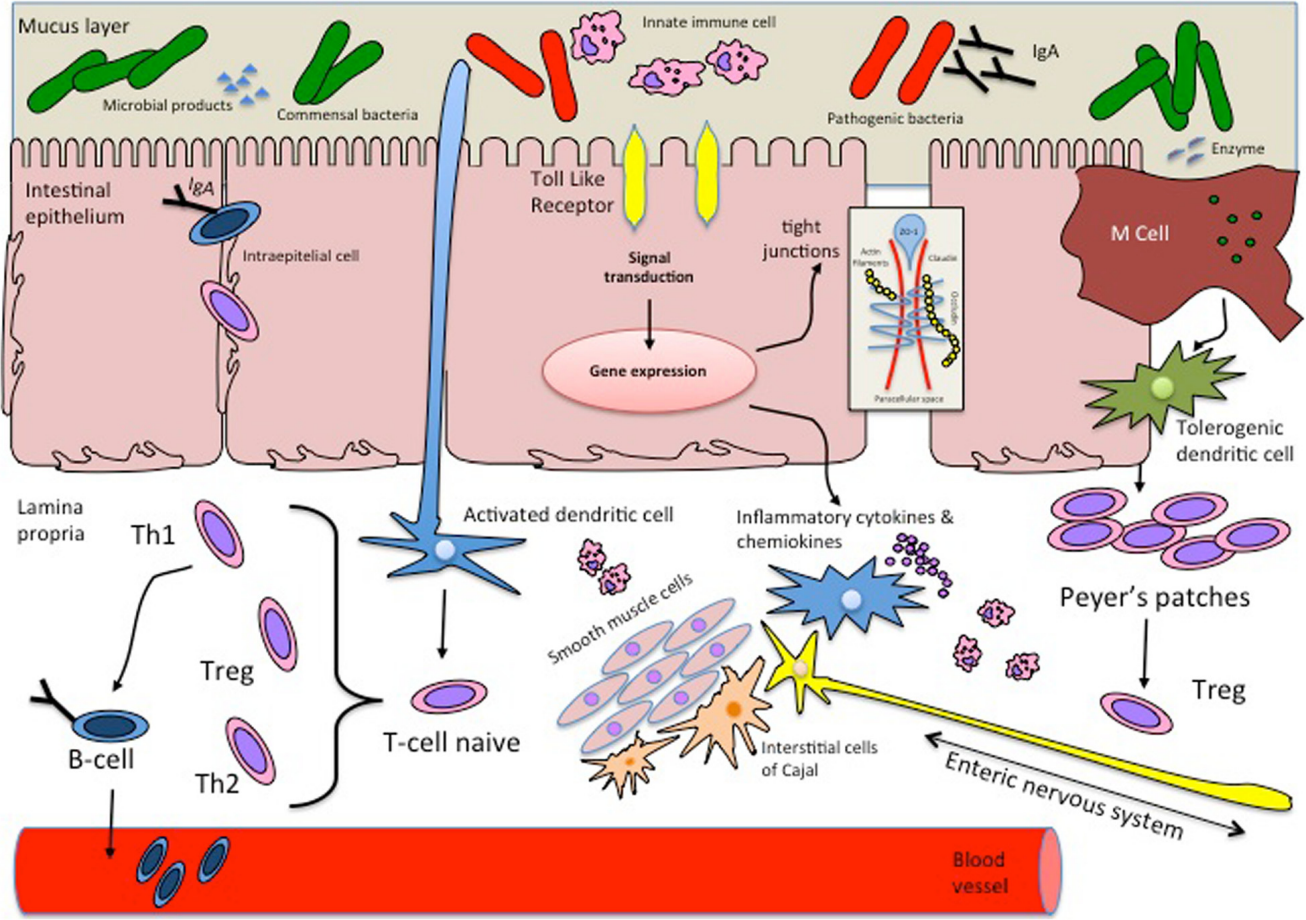
**Intestinal function and microbiota.** Commensal bacteria inhabiting the human intestine participate in the development and maintenance of gut immunologic, sensory and motor functions. Under normal conditions, the gastrointestinal tract provides a stable habitat for commensal bacteria that supports its structural and functional integrity. The ENS influence the gut directly with the activity related to the contraction and indirectly influencing the cells of the gut immune system. The functional bidirectional interaction act via neuroimmune peptide receptor on immune cells and on several receptor for immune mediators expressed on enteric nerves. Immune cells release mediators in response to neural stimuli.

The gut interacts with intestinal bacteria to mature protective mechanisms against harmful molecules (via improving barrier gut functions, motility and immune stimulation) and appropriate, non-exaggerated responses versus commensal bacteria and nutrients (via immune-modulation and immune-tolerance) [20]. The mechanisms of this interaction between host and bacteria are increasingly being unrevealed. An aberrant bacterial colonization may be a coexisting factor in feeding intolerance in newborn. The exact effects of bacterial colonization in pathophysiology of feeding intolerance is based on the post-natal uncorrect ontogenesis of the intestinal barrier, of the immune responses and of sensori-motor functions of the gut [21] Figure 1.

### Microbiota modulation on development of gastrointestinal functions

#### Microbiota and intestinal barrier function

Microbial intestinal content plays an important part in the evolution of gastrointestinal structure via direct interaction with mucosal cell, immune cells and neuronal endings. To support this statement, some studies have shown structural aberration affecting free-germ mice: greatly enlarged cecum, reduced intestinal surface area, decreased epithelial cell turnover, smaller Peyer’s Patches and disordered gut-associated lymphoid tissue and smaller villous thickness [22,23]. This morphological consequences of growing up germ-free result in gastrointestinal functional disorders.

Thus, bacteria regulate development of the intestinal barrier and, consequently, its functions. Studies have shown that certain bacteria (*E. coli, Bifidobacterium, Lactobacillis*) increase intestinal epithelial cell survival by inhibiting the activation of the epithelial cell pro-apoptotic pathway associated with pathogenic bacteria [24]. The commensal microbiota is also involved in maintenance of barrier function inducing an increased epithelial cell proliferation and enhancing intestinal epithelial integrity, through translocation of the tight junction proteins and up-regulation of genes involved in desmosome maintenance [25]. Finally, commensal bacteria regulate development of intestinal villus vascular architecture [26].

Surface enterocytes can recognize bacterial products via a highly conserved family of pathogen-associated molecular pattern (PAMP) receptors called Toll-like receptors (TLR). Each of these receptors recognizes a specific bacterial product.

Binding of any of this receptors leads to activation of nuclear factor-κB (NF-κB), a group of proteins that start the transcriptional process of a wide amount of genes.

Despite commensal bacteria interference with pathogenic infections through effects on intestinal mucosa structure, the prevention of colonization by pathogens is achieved in large part by competing for nutrients and receptors, by production of anti-microbial compounds and by triggering the expression of multiple cell-signaling process that can limit the release of virulence factors.

The intestinal mucosa barrier is composed by both non-specific defensive mechanisms, such as intestinal motility, mucus secretion, gastric acids and pancreatic enzymes, and specific immune mechanisms that prevent the transit of external and unprocessed antigens across the gastrointestinal barrier [27].

The gut-barrier faces important challenges: to prevent pathogens and harmful elements of the gut lumen from entering into the internal environment of the host; to allow the absorption of nutrients; and finally to promote passage of molecules and information between gut lumen and the components of the endocrinal, neuronal, immunal routes implicated in maintenance of homeostasis.

Exposed to trillions of microbes and countless food antigens of the gut lumen, the intestinal mucosa is incessantly tasting luminal elements and promoting molecular modification at its frontier to respond in different way to commensal bacterial or to pathogens.

The early microbial composition of the human gastrointestinal tract has long-lasting functional effects, affecting the postnatal immune system development and an aberrant early colonization that may provoke difficulty in the capacity to distinguish potentially dangerous from harmless antigens. A reduced or a abnormal microbial colonization during first months of life would also provoke a slower postnatal maturation of epithelial cell barrier functions with a consequential altered permeability that facilitates the invasion of pathogens and foreign or harmful antigens [28].

An abnormal microbial colonization could finally lead to mucosal inflammation that plays a pivotal function in the development of feeding intolerance [29].

#### Microbiota and gastrointestinal mucosal immunology

The human immune system includes the innate immunity, that has a standardized response to all adverse agents, and the adaptative immunity, that recognize specifically each microorganism and have a specific response and memory. With regards to newborns, we have also to mention the immunity passively acquired by trans-placental transport of maternal immunoglobulin G in utero and from human milk secretory IgA antibody after birth. Since the intestinal mucosa constantly exposed to antigenic stimulation, the protective function of the gut requires different factors to stimulate either innate and adaptive immune response, in a complex and well regulated net of tolerance-inducing mechanisms residing in the GALT (gut associated lymphoid tissue), the most extensive lymphoid system of the human body.

GALT is formed by both inductive (Peyer’s patches) and effector sites (lamina propria and sub-epithelial cells). It works as a containment system that prevents the transit of external and unprocessed, potentially harmful, antigens across the intestinal barrier but is also constantly in contact with the microbiota and with food antigens.

Germ free studies have revealed that the microbiota is one of the most important factor for the development of the GALT [30]. GALT prevents potentially harmful intestinal antigens through polymeric immunoglobulin A (IgA) secretion and modulates the tolerance versus luminal antigens through processes that involve specific cytokine and peculiar population of cells. This fundamental enteric function, known as *oral tolerance*, is based on the interaction between the luminal content, the intestinal epithelium and the tolerogenic dendritic cells (DCs) from mesenteric lymphonodes of the GALT [31]. Oral tolerance is able to avoid inflammatory response against food proteins and self aggression against the host’s own resident intestinal bacterial microbiota through the establishment of a tolerogenic mechanism on CD4+ T cell naïve that suppress the expression of T effector cells (Th1 and Th2) and stimulate the expansion of regulatory T cells (Tregs) by secreting cytokines such as IL-10 and TGFβ. M cells and DCs of the GALT can in fact sample the microbial milieu: M cells efficiently take up in lumen and transport in the lamina propria a variety of microorganisms and antigens via active vesicular transport across the epithelium. Lamina propria DCs process antigens from intestinal lumen by sending dendrites between enterocytes and present them to T naïve cells.

In the lamina propria, isolated and epithelium-associated lymphoid follicles are proposed to be local sites in gut for interaction between subepithelial antigen-presenting cells, antigens and lymphocytes. In this setting, DCs modulate immune responses through activation of signaling events leading to improve expression of factors, such as cytokines and chemokine that recruit and regulate the phenotype and functions of immune T cells [32]. Antigens are presented by dendritic cells in the contest of MHC class II molecules to naïve T lymphocytes. Intestinal responses of the naïve T cells to these either food or bacterial signals are generally described in terms of two classes of CD4+ T cells, defined by their cytokine production: T helper type 1 that modulate cell-mediated immunity by secreting INFγ and TNFα; and T helper type 2 that modulate humoral immunity by secreting IL-4 and IL-6. In a non-diseased state there is a tight regulation of these cytokines.

The immature immune system of newborn is known to have a Th2 bias. The postnatal gut colonization makes an appropriate shift towards a Th1 response that results in a balance of the system. Microbial colonization influences toll-like receptors on gut immune cells that recognize PAMPs and modulate both intestinal innate and an appropriate adaptive immune response, according to the characteristics of microbial strain (commensal or pathogen).

Different lactic acid bacteria as *Lactobacillus* and *Bifidobacterium* have been shown to determine a proportioned T helper cell response, forbidding a T cell unbalance (Th2 > Th1 or Th1 > Th2) that may conduce to clinical disease [33].

These findings demonstrate that the intestinal microflora and its qualitative differences in composition might affect immunologic homeostasis. The balance between microbiota, immune response and tolerance mechanisms is fundamental for a healthy intestine, and inappropriate relationship due to an abnormal colonization may result in feeding intolerance in early postnatal life and in gastrointestinal disease in childhood.

#### Microbiota-gut-brain-axis

In the past few years, growing evidence supports the importance of microbiota in the maturation and modulation of gut sensorimotor functions. Gut sensory and motor function are under control of the gut-brain axis, a complex bidirectional communication system that exists between the central nervous system and the gastrointestinal tract. Microbiota can interact with this axis emitting and receiving a multiplicity of signals to and from the brain. This has been reflected in the form of a revised nomenclature to the more inclusive brain-gut-microbiota axis and a sustained research effort to establish how communication along this axis contributes to both normal and pathological condition [34].

The brain can influence microbiota composition indirectly through modifications in gastrointestinal motility, secretion, and intestinal permeability, or directly, via citokines released into the gut lumen from enterochromaffin cells, neurons and immune cells of lamina propria. Through the removal of exuberant bacteria from the lumen, intestinal motility is considered one of the most important control systems of the intestinal microbiota, On the other hand, enteric microbiota also play a role in the development and sustenance of both sensory and motor gut functions, through communication with the brain that occurs indirectly through interaction with epithelial-cell and receptor-mediated signaling and directly through stimulation of neuronal cells in the lamina propria, when intestinal permeability is increased [35].

Integral to these communications are enterochromaffin cells, which serve as bidirectional transducers that modulate interrelationship between the gastrointestinal lumen and the nervous system [36]. Enterochromaffin cells are innervated by the sensory fiber of vagus nerve. Gut microbiota influence on enterochromaffin cells suggest a role in the regulation of visceral pain. The correct mechanisms of action of such effect currently remain unclear. Furthermore, evidence for combination of neural, immune and endocrine effects emerges from studies [37].

An immature ontogenesis of the bidirectional interrelation between the enteric microbiota and the nervous system, due to an aberrant colonization after birth, could affect the pathophysiology of feeding intolerance in preterm newborn and of different childhood functional gastrointestinal disease later in life [38].

#### Microbiota and feeding intolerance in preterm newborn

Disturbance of normal gastrointestinal ontogenesis, early postnatal stress, different pattern of gastrointestinal colonization, changes in the microbiota induced by infection or early use of antibiotics in Neonatal Intensive Care Unit (NICU), or other events, perturb physiologic inflammation and gut physiology, resulting in an aberrant activation of intestinal peristalsis and gut-brain axis [39]. Aberrant intestinal functions development are the major determinant for feeding intolerance (FI), a major problem in NICU. FI is defined as the inability to digest enteral feedings and may be considered as a predictive value for a developing NEC [40]. FI occurs most commonly in very low birth weight (VLBW) infants, indicating a deficiency in the developmental pattern of gastrointestinal tract with decreasing gestational age (GA). Newborns require structural and functional maturation of gastrointestinal tract for the digestion and absorption of the nutrient elements from colostrum and breast milk. A complete intestinal motor function development includes suck - swallow coordination, gastroesophageal sphincter tone continence, adequate gastric emptying and intestinal peristalsis. Term newborns are able to acquire adequate quantities of nutrients to promote the rapid growth that occurs shortly after birth. However, half of preterm infants are delayed in achieving full enteral feeding volumes and present reflux, gastric residual and constipation due to delayed gastric emptying, prolonged intestinal transit, abdominal distension, and delayed passage of meconium, all of which reflex gastrointestinal functions immaturity [41]. There are few studies available about the fetal ontogenesis and the neonatal early adaptation of motility and mucosal barrier functions of the human gut [42-44]. Functional components of the human gastrointestinal tract do not evolve simultaneously: in facts, althought anatomical differentiation of human gut is usually achieved within 20 weeks of gestation, the functional maturation is postponed over time and require an organized peristalsis and a coordinated sucking and swallowing reflexes, that are not extabished, respectively, until 29–30 weeks and 32–34 weeks of gestations [45]. As illustrated before, the early composition of the intestinal microbiota at birth can influence the correct ontogenesis of gut barrier, motor and immune functions through a complex neuroendocrine cross-talk [46]. Thus, an appropriate colonization of the gastrointestinal tract after birthis likely to play an important role in the final development of gut functions. Premature infants have an abnormal colonization, tend to colonize with fewer bacteria, are routinely administered antibiotics, are often born via caesarian section, and are exposed to highly pathogenic institutional organisms [47,48]. Examining the intestinal bacteria present in premature infants may be an important determinant in the pathogenesis of feeding intolerance and necrotizing enterocolitis (NEC) [49]. Using advanced technologies and stool samples studies have shown that infants who develop NEC have significantly less bacterial diversity in their intestinal microbiota with presence of certain pathogenic bacteria and the lack of protective bacteria [50,51].

Exposure to “non-beneficial” microorganisms and antibiotics early in life may result in immune dysregulation, aberrant barrier functions, and alterated gut sensorimotor functions that, in susceptible individuals, may lead to some disease states after birth or later in life. Efforts to optimize the intestinal microbiota colonization at birth in neonates who are born by caesarean delivery, born preterm, exposed to antibiotics and/or fed with infant-sterile-formulas, have increased the interest in early probiotics supplementation. Introducing probiotic to preterm infants has been postulated to enhance enteral feeding, prevent NEC and avoid overgrowth of pathogenic organism. Probiotics are live microbes that provide health benefits to the host when dispensed in adequate doses. Many strains that are part of the human intestinal microbiota could be considered as potential probiotics but microorganisms used in prevention and treatment of pediatric clinical diseases are typically members of the genera *lactobacillus* and *bifidobacterium*, following the natural evolution of the microbial colonization in a healty term breast-fed baby.

## Conclusion

The intestine serves as a vast interface between our internal milieu and external environments. Evidence is rapidly accumulating that the microbes residing within the intestinal tract play major roles in the ontogenesis of the immune system, and interact with the gut as well as the central nervous systems.

An aberrant microbial colonization with consequential immaturity in development of immune and neuronal pattern of gastrointestinal tract may be a coexisting cause of feeding intolerance. Abnormal colonization should constantly be kept in mind as an important environmental factor that predisposes to disease also later in life.

It is emphasized that the perinatal period most probably corresponds to a critical time at which “set points” are imprinted. More needs to be known about normal and healthy colonization patterns in newborns to promote these patterns and to avoid perturbations that result in lifelong disease.

## Competing interests

The authors declare that they have no competing interests.

## Authors’ contributions

All the authors contributed to the review and were involved in writing, revising and approving the final draft of the manuscript.
